# Malignant Œdema

**Published:** 1896-03

**Authors:** 


MALIGNANT ŒDEMA (from the same article). I saw one case in a horse in Windhoek in May, which I diagnosed as malignant œdema on the ground of general symptoms, without bacteriological examination. The horse, a young and restless animal from 2 to 2½ years old, was left hobbled in the pasture during the night, as is the custom in the country. The hobbling was done in this case as usual, with a strap about as thick as one's finger. This strap is fastened in two loops around the middle ankle bones of the front legs, in the middle between the ankle and fetter-joint, and drawn together until the distance between the legs is about the length of one's hand. The horse can take only very small steps and usually prefers to spring forward with both forelegs together. The strap is made mostly of untanned leather, is hard and unyielding, and cuts in easily. This had taken place in the case under consideration The middle of the backside of the ankle on both forelegs was galled in spots of about 2 cm. in breadth and 5 cm. in length. On the near front foot the excoriation was considerably longer. and somewhat deeper. From this point up the whole foreleg as far as the shoulder-joint was swollen to double its natural size. It was also very hot and extremely sensitive to pressure. The horse was wretched, had no appetite, his ears and hoofs were hot, and he showed generally a high temperature. From the excoriation there was dropping a thin discolored filthy liquid filled with air-bubbles. The swelling had almost the consistency of dough. Emphysema could only be felt very close to the wound. The sickness had existed several hours when I saw the animal, *i. e.*, it had been noticed early in the morning and I did not arrive until 10 o'clock. The treatment consisted in long, deep incisions and bandaging with creolin, which was renewed twice daily. On the next day the swelling had already decreased very considerably, and during the two following days decreased so as to be confined to the immediate neighborhood of the excoriation wound, while the secretion had become quite natural. The incisions healed up without difficulty. I saw the animal several months later in the best of health. Excepting this one case, I have never heard or read of another. I was also unable to find out what the natives call this disease. In England it bears the name of malignant œdema. It seems to occur very seldom, perhaps on account of the dry and loose nature of the soil in South Africa and the small extent to which manure is put upon the land. At least a great deal supports the view that there is a connection between the amount of dampness and manure in the earth on the one side and the bacillus of malignant œdema on the other, for the bacillus is found most often in garden earth which has been heavily manured and constantly sprinkled.

